# The dorsal lateral geniculate nucleus and the pulvinar as essential partners for visual cortical functions

**DOI:** 10.3389/fnins.2023.1258393

**Published:** 2023-08-30

**Authors:** Christian Casanova, Leo M. Chalupa

**Affiliations:** ^1^School of Optometry, University of Montreal, Montreal, QC, Canada; ^2^School of Medicine and Health Sciences, The George Washington University, Washington, DC, United States

**Keywords:** vision, transthalamic cortical pathways, receptive fields, lateral posterior nucleus, thalamus

## Abstract

In most neuroscience textbooks, the thalamus is presented as a structure that relays sensory signals from visual, auditory, somatosensory, and gustatory receptors to the cerebral cortex. But the function of the thalamic nuclei goes beyond the simple transfer of information. This is especially true for the second-order nuclei, but also applies to first-order nuclei. First order thalamic nuclei receive information from the periphery, like the dorsal lateral geniculate nucleus (dLGN), which receives a direct input from the retina. In contrast, second order thalamic nuclei, like the pulvinar, receive minor or no input from the periphery, with the bulk of their input derived from cortical areas. The dLGN refines the information received from the retina by temporal decorrelation, thereby transmitting the most “relevant” signals to the visual cortex. The pulvinar is closely linked to virtually all visual cortical areas, and there is growing evidence that it is necessary for normal cortical processing and for aspects of visual cognition. In this article, we will discuss what we know and do not know about these structures and propose some thoughts based on the knowledge gained during the course of our careers. We hope that these thoughts will arouse curiosity about the visual thalamus and its important role, especially for the next generation of neuroscientists.

## 1. Foreword

Much has been accomplished since the first recordings were made from the mammalian dorsal lateral geniculate nucleus (dLGN) by Barlow, Fitzhugh and Kuffler in 1957. These pioneering investigators found that the visual receptive field properties of dLGN neurons were in many ways similar to those reported earlier for retinal ganglion cells by Kuffler (1953). These early studies, as well as the observations of Hubel (1960), laid the groundwork for the long-maintained viewpoint that the dLGN functions largely to relay visual information from the retina to the primary visual cortex. Indeed, this notion can still be found in some neuroscience and medical textbooks today. Similarly, interest in the pulvinar, the largest thalamic nucleus in mammals, originally stems from its connectivity with the mesencephalon and the cortex. The pulvinar was also often considered as a relay nucleus, providing a route for signals from the superior colliculus, a structure involved in the control of fixation and eye movements, to the visual cortex.

The problem with the term “relay” is that it suggests that information is transmitted without significant modification or transformation. We now know that this is not the case. Indeed, while the precise roles of the dLGN and the pulvinar in visual and extra-visual functions are yet to be established, a rather substantial body of literature has clearly refuted the notion that these are “relay” nuclei. Our intent in this paper is to provide an overview of some of the key studies in this field and to propose some thoughts as to how this aspect of visual neuroscience could be advanced in future work.

## 2. The dorsal lateral geniculate nucleus

The dLGN is undoubtedly the most studied subcortical region of the visual brain (Werner and Chalupa, 2004). In mammals with front-facing eyes, this knee-shaped structure is organized in distinct layers each receiving signals from one of the two eyes. Thus, information from the two eyes remains separate until it reaches the primary visual cortex where binocularity is formally established. Hubel (1960) was the first to record visual activity of optic tract units as well as those of dLGN neurons in the same animal preparation, which led him to conclude that the organization of the receptive fields of dLGN neurons are strikingly similar to those of retinal ganglion cells. Later, Enroth-Cugell and Robson (1966) discovered three parallel retinal pathways (X, Y and W) that differ in spatial and temporal resolution, contrast sensitivity and conduction velocity. The latter were subsequently revealed in primates (Kaplan and Shapley, 1982), and it is now well established that the four dorsal layers (parvocellular) of the dLGN receive signals about form and color from X-type ganglion cells and the two ventral layers (magnocellular), receive signals about movement from Y-type ganglion cells. Ventral to each of the magno- and parvocellular layers lie the koniocellular layers, which contain a heterogenous group of neurons whose functions remain to be fully determined. While the main emphasis of these early studies was the role of the retinal input in the organization of dLGN receptive fields, subsequent anatomical studies showed that only about 10 percent of the synaptic contacts onto dLGN neurons derive from the retina. The astonishing conclusion from the anatomical evidence was that most of the input to the dLGN is from non-retinal sources, mainly the visual cortex and various brainstem nuclei (Jones, 1985).

**Figure 1:**
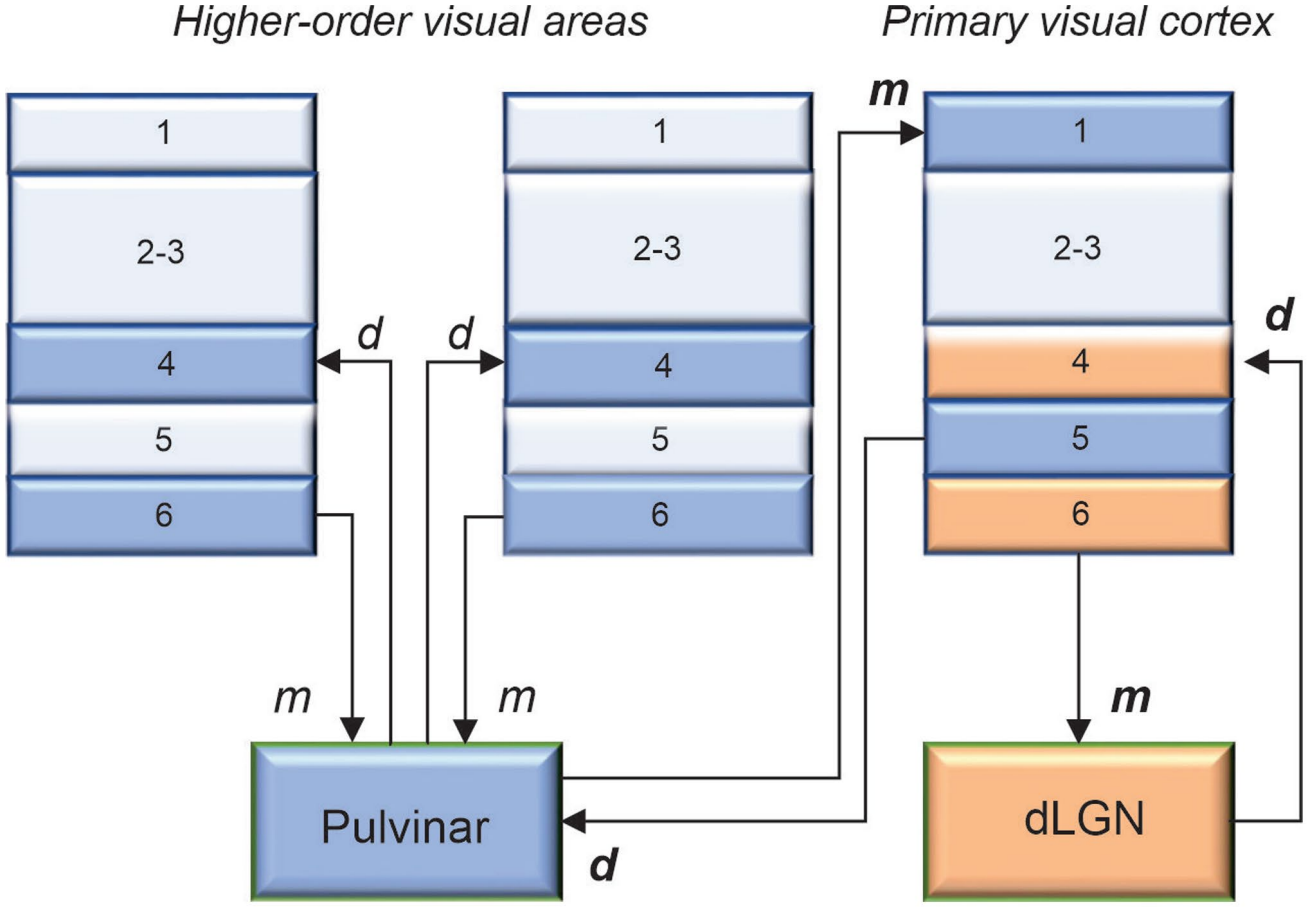
The main predicted laminar connections involving the pulvinar are based on the general rules proposed by Jones (1985), with additional constraints advanced by Crick and Koch (1998). The geniculo-cortical pathway is provided for comparison. Cortico-cortical projections are omitted for clarity. *d*: drivers, *m*: modulators. When in bold, *d* and *m* were confirmed functionally.

How is it that the retina, which represents only about 10% of the dLGN afferents, has the strongest functional input? The answer, in part, comes from the work of Sherman and Guillery (1998). Based on several morphological and functional criteria, these authors have identified two types of inputs: drivers and modulators (type 2 and type 1 projections, respectively). Essentially, drivers determine the main properties of the activity of their target cells, whereas modulators provide contextual modulation of the recipient neuron’s activity. Studies have shown that retinal ganglion cells provide a driver input to dLGN neurons, which in turn send driver signals to layer 4 neurons in V1. In return, layer 6 neurons of V1 projecting to the dLGN exert a modulatory influence (Figure 1). These laminar patterns are in accordance with the assumption made by several authors (cf, Jones, 1985), that drivers and modulators terminate and originate from distinct cortical layers. For bottom-up projections, thalamocortical terminals ending in layer 4 are drivers, while those ending in layer 1 are modulators. For top-down projections, cortico-thalamic cells in layer 5 provide driver signals, while those lying in layer 6 send modulatory signals. Thus, visual processing in the dLGN is continuously influenced by patterns of activity occurring in V1. By considering the visual response latency and conduction latency of corticogeniculate neurons, Briggs and Usrey (2007) estimated that cortical feedback signals can reach the dLGN within ∼50 ms of the presentation of a visual stimulus. It is acknowledged that these signals have little effect on the spatial characteristics of dLGN receptive fields inherited from retinal inputs or on their “sharpness” (Murphy and Sillito, 1987; Sillito and Jones, 2002; Webb et al., 2002). However, there is mounting evidence that the visual cortex enhances response precision of dLGN neurons by reducing their gain variability, thereby increasing their information coding capacity (Hasse and Briggs, 2017; Murphy et al., 2021; Spacek et al., 2022).

So, what does the dLGN do besides transferring retinal signals to the cortex? In most textbooks, one will learn that the dLGN is gating information according to the state of vigilance. Depending on the influence of signals from the reticular activating system of the brainstem, dLGN neurons will discharge with regularly spaced action potentials (tonic mode, often associated with arousal) or with clusters of spikes (burst mode, associated with sleep), dissociating the dLGN from external signals. We invite the reader to consult the comprehensive reviews of Weyand (2016) and Ghodrati et al. (2017), who propose several functions of the dLGN reflecting the numerous non-retinal inputs that allow the dLGN to transform retinal signals in dynamic ways. We would like to mention here two notable facts. The tonic-burst mode of dLGN neurons is not solely related to arousal as previously thought. It has been shown that V1 feedback projections shift the firing mode of geniculate neurons between burst and tonic patterns (Sherman, 2001). Alitto et al. (2019) showed that retinogeniculate communication is enhanced during the burst mode and visually evoked thalamic bursts, thereby augmenting retinal signals transmitted to cortex. Computational models suggest that burst neurons can encode stimulus features similarly to their tonic counterparts (Elijah et al., 2015; Alitto et al., 2019). Thus, by transitioning between the two modes in a dynamic way, cells in dLGN may allow the most relevant information to be transferred to the visual cortex during normal waking behavior. Another interesting fact is that the temporal aspect of retinal and dLGN responses differ considerably. This has led to the proposal that dLGN neurons improve visual coding by reducing redundancy through temporal decorrelation of the retinal signals, much in the same way that the retina improves efficiency by spatially decorrelating incoming images (Truccolo and Dong, 2001; Tan and Yao, 2009; Alexander et al., 2022).

Clearly, the dLGN is more than a “simple” relay nucleus. The retinal signals inputs to this thalamic structure are modulated by non-retinal inputs, such as those originating from the visual cortex, the mesencephalon, the thalamic reticular nucleus, the extraocular muscles, and the brainstem. The dLGN is not merely a gate that opens and closes, nor simply a linear filter. Its plays a fundamental role in regulating information transmission to the visual cortex as a function of the activity taking place in subcortical and cortical areas, thereby enhancing the message being transferred to the first site of cortical computation, i.e., V1 (Fisher et al., 2017). But is the dLGN involved in higher-level processing as proposed by some authors (Zabbah et al., 2014; Ghodrati et al., 2017), or should we turn our attention to the pulvinar nucleus for that?

## 3. The pulvinar nucleus

Situated next to the dLGN (the “knee”) is the pulvinar nucleus (the “cushion”). The pulvinar lies over the dorsolateral posterior thalamus and runs along the medial edge of the dLGN. The size and differentiation of the pulvinar increased strikingly during evolution, in parallel with the neocortex (Chalfin et al., 2007; Kaas and Baldwin, 2019). Thus, it is the largest visual nucleus of the thalamus in primates and cats, which have been the two most studied species in pulvinar research. In both species, it has established extensive reciprocal connections with all visual cortical areas (see Casanova, 2004). Six subdivisions have been identified in the pulvinar of primates, including humans, and several representations of the visual field have been delineated (Bender, 1981; Arcaro et al., 2015; DeSimone et al., 2015; Baldwin et al., 2017). In the cat, the lateral posterior-pulvinar complex is generally subdivided into three main regions (up to five have been proposed, Hutchins and Updyke, 1989), each containing a coarse representation of the contralateral visual field: the lateral and medial parts of lateral posterior nucleus (LPl and LPm) and the pulvinar *per se*, also named the striate-, tecto-, and retino-recipient zones of the complex, respectively (Berson and Graybiel, 1983; Abramson and Chalupa, 1985). The nomenclature of this nucleus across species is rather confusing. In animals like rodents and rabbits, it is named the lateral posterior (LP) nucleus. In cats, the two main subdivisions of the LP are also considered as the homolog of the primate pulvinar, while a portion of the cat pulvinar has been considered as part of the geniculate complex (Mason, 1978). A reevaluation of the terminology is necessary. For clarity, only the term “pulvinar” will be used thereafter.

While there is a plethora of studies on the dLGN and the primary visual cortex, fewer have focused on the pulvinar, particularly on the properties of its neurons. This is undoubtedly due to the difficulty of documenting the visual response properties of pulvinar cells, which are much more capricious than those of the dLGN and more sensitive to anesthetics. Interest in the pulvinar stems from the discovery of the existence of two visual systems (Schneider, 1969): one for discrimination (retina-dLGN-visual cortex) and the other for localization (retina-superior colliculus). At the time, some laboratories such as that of Donald B. Lindsley (Chalupa et al., 1972), focused their work on the pulvinar, as signals from the superior colliculus must pass through the pulvinar to reach the visual cortex, a research theme that has been addressed recently by Beltramo and Scanziani (2019). Unlike the dLGN, which receives its main visual inputs from the retina, the pulvinar gets its visual signals mainly from the neocortex and the mesencephalon. The strong cortical input is reflected in the response properties of its neurons, which remarkably resemble those found in the visual cortex (Bender, 1982; Chalupa and Abramson, 1988; Casanova et al., 1989; Chalupa and Abramson, 1989). Essentially, pulvinar cells have large receptive fields, largely encompassing those of V1 neurons, with a complex cell-like organization (Casanova et al., 1989; Piche et al., 2015). In contrast to dLGN neurons, most pulvinar cells are binocular, sensitive to retinal disparity, and selective for stimulus orientation and direction of motion. Moreover, in cats (as well as in humans), pulvinar neurons can signal the true direction of motion of complex stimuli such as plaid patterns and random dot kinematograms (Merabet et al., 1998; Dumbrava et al., 2001; Villeneuve et al., 2005; Thompson et al., 2012; Villeneuve et al., 2012). This level of computation is generally described in higher-order cortical areas only, further suggesting that the pulvinar actively participates to the processing of visual signals transferred along the cortical hierarchy. Pattern-motion neurons were mostly found in the LPm subdivision of pulvinar. Differences between the response properties of the two main subregions of the pulvinar, the LPl and LPm, have been described and likely reflect the predominance of V1 and collicular inputs targeting the LPl and LPm, respectively (Chalupa and Abramson, 1989; Piche et al., 2015). The interest of researchers toward the pulvinar was increased by the discovery that the activity of a subset of cells is linked to visual attention (Petersen et al., 1985). Subsequent studies on behaving monkeys and humans (normal and with lesions) have indeed confirmed that this nucleus is involved in visual attention, visuo-motor functions, target selection among distractors and feature binding (Chalupa et al., 1976; Ward et al., 2002; Danziger et al., 2004; Shipp, 2004; Zhou et al., 2016; Fiebelkorn et al., 2019). In recent years, given it connectivity with the limbic system, the pulvinar has been linked with the visual processing of fearful stimuli (Ward et al., 2005; Ren and Tao, 2020). Moreover, pulvinar is considered to play an important role in unconscious vision or blindsight (Cowey et al., 1994; Fox et al., 2020).

**Figure 2:**
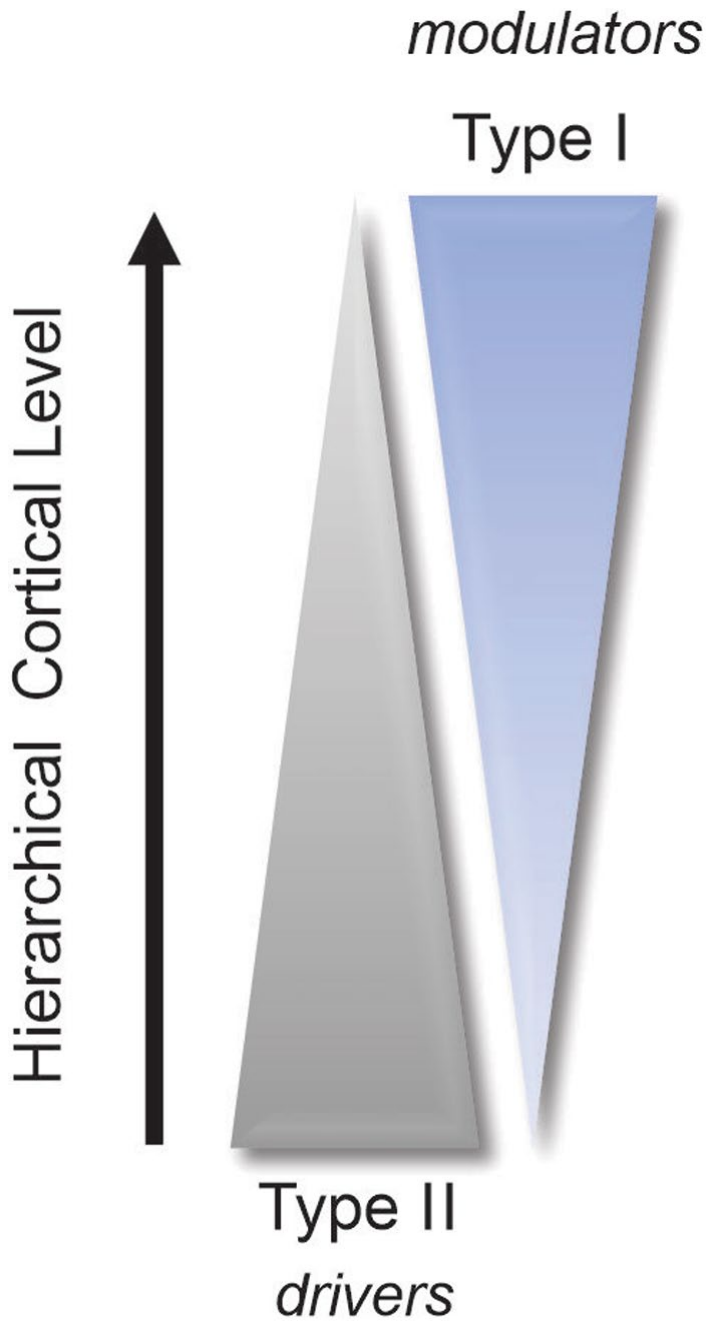
Simplified diagram illustrating the increase in modulator terminals (type 1) and the concomitant decrease in driver terminals (type 2) along the cortical hierarchy (refer to Abbas Farishta et al., 2020).

This array of functions associated with the pulvinar not only reflects the extensive connectivity of this thalamic complex with the neocortex but also illustrates why researchers are still struggling to assign a clear role to the pulvinar. This is why we must return to the roots and determine the nature of the signals being exchanged between the pulvinar and the visual cortex. As mentioned above, given its extensive reciprocal connectivity with the visual cortex, the pulvinar is in an exceptional position to regulate cortical processing along the visual hierarchy, both in the feedforward and feedback directions. For decades, the perception of external stimuli was considered to result solely from the processing of thalamic signals through direct cortico-cortical connections between hierarchically organized areas. We must now consider the fact that, besides direct communication between cortical areas through cortico-cortical connections, indirect communication through cortico-pulvinar-cortical projections also occurs (Figure 1). Through these *transthalamic pathways*, each cortical area of the visual cortex is potentially only one thalamic synapse away from another cortical area, allowing a rapid modulation of cortical activity according to external signals and internal computations. What do we know about these transthalamic cortical pathways, which has been the subject of research by several laboratories (e.g., Rockland, 1996; Baldauf et al., 2005; Huppe-Gourgues et al., 2006; Rockland, 2019). We know that V1 provides a strong driver input to the pulvinar from layer 5 complex cells (Casanova, 1993). Permanent or transient inactivation of V1 results in an almost total disappearance of visual responses in the striate-recipient zone of the pulvinar in cats and primates, indicating that V1 is necessary for establishing the fundamental representation of the visual world in the pulvinar (Bender, 1983; Casanova et al., 1997). The contribution of other areas to the subregions of the pulvinar is still not fully understood. Studies have shown that pattern-motion neurons in the LPm of cats only disappear when both V1 and the ectosylvian cortex are lesioned (Merabet et al., 1998). Recently, Abbas Farishta et al. (2020) revealed that the ratio of type 1/type 2 terminals (thus, putatively, the modulator/driver ratio) from layers 5 and 6 neurons terminating in the pulvinar increases along the cortical hierarchy (Figure 2). This organizational scheme predicts that higher-order visual cortical areas will primarily modulate activity in the pulvinar. A theoretical model based on this organization suggests that the pulvinar shows a bistable spiking activity, oscillatory or regular asynchronous spiking, whose responses are gated by the different activation of cortico-pulvinar projections from lower to higher-order areas (Cortes et al., 2021), but this has yet to be demonstrated functionally.

A greater number of studies have explored the impact of the pulvinar on the visual cortex. However, during some of these studies, the understanding of drivers and modulators was either unknown or in its early stages. As a result, the visual stimuli and experimental approaches were not specifically designed to differentiate between these two types of signals (e.g., Minville and Casanova, 1998). Vanni et al. (2015) discovered that direct stimulation of the pulvinar and dLGN in tree shrews produced distinct spatiotemporal profiles of voltage-sensitive dye responses in the visual cortex. Stimulation of the dLGN resulted in rapid, strong, and localized responses in the primary visual cortex, characteristic of a driver input. On the other hand, pulvinar activation evoked only weak and diffuse responses in the same area, indicative of a modulatory input. Interestingly, pulvinar stimulation evoked fast and robust responses in higher-order visual areas. This finding supports the notion that the pulvinar predominantly projects to layer 1 of V1 (modulatory input) and layer 4 (driver input) in areas beyond V1 (Abramson and Chalupa, 1985). However, Logothetis et al. (2010) reported no compelling differences between the positive blood oxygen level-dependent (BOLD) signals in V1 and extrastriate cortical areas when the pulvinar was electrically stimulated in anesthetized monkeys. Subsequently, Roth et al. (2016) revealed a distinct organization of thalamic input from the dLGN (circumscribed and visuotopically organized) and the pulvinar (more distributed) in the primary visual cortex of mice. This suggests again that the pulvinar is more likely involved in the contextual modulation of activity in V1.

In a different set of recordings conducted on macaque monkeys, Saalmann et al. (2012) demonstrated that the pulvinar is capable of synchronizing activity between cortical areas based on attentional allocation. This finding aligns with a study in cats showing that the pulvinar modulates the oscillatory information between areas 17 and 21a in gamma and alpha bands for feedforward and feedback processing, respectively (Wrobel et al., 2007; Cortes et al., 2020). Together, these findings indicate that the pulvinar is involved in the mechanisms underlying oscillatory communication along the visual cortex. The role of the pulvinar in attentional processing was further suggested by Zhou et al. (2016), who showed that inactivation of the macaque pulvinar leads to a decrease in attentional effects on firing rates and gamma synchrony and coherence of neurons in area V4. Additionally, a more recent study conducted in ferrets demonstrated that fluctuations in pupil-linked arousal result in dynamic changes in frequencies along the thalamocortical network (Stitt et al., 2018). During low arousal states, synchronized activity between the pulvinar and the posterior parietal cortex was characterized by alpha oscillations, while higher arousal states exhibited theta frequency bands. The authors proposed that the pulvinar may act as a gate or suppressor of incoming sensory information in the early stages of the visual cortex during low arousal but facilitate visual exploration and attentional selection during increased arousal. A similar pattern of synchronization was observed in recordings between the mediodorsal pulvinar, the frontal eye fields (FEF), and the lateral intraparietal region (LIP) of macaques by Fiebelkorn et al. (2019). According to these authors, the pulvinar may gate communication between cortical areas by aligning the phases of their oscillatory responses.

De Souza et al. (2020) conducted a study to examine the impact of the pulvinar on the contrast response function of neurons in two hierarchically distinct areas of the cat visual cortex. Their findings highlight the complexity of the functional relationship between the pulvinar and the cortex. In the primary visual cortex (V1), pulvinar inactivation resulted in a modest decrease in the response gain of neurons to contrast. This effect was less pronounced than the near-complete suppression of activity observed in the supragranular layers of V1 in prosimian primates when the pulvinar is silenced (Purushothaman et al., 2012). Surprisingly, contrary to expectations based on anatomical evidence, pulvinar inactivation led to a significant increase in response gain in the majority of neurons in area 21a (often considered the homolog of primate area V4), with only a subset of cells exhibiting changes in contrast gain. Similar increases in response amplitude were also observed in area V2 of Cebus monkeys when the pulvinar was inactivated (Soares et al., 2004). The study by De Souza et al. (2020) demonstrates that the pulvinar can influence functions across the visual cortex through the modulation of neuronal activity. This suggests that the mechanisms underlying the transthalamic flow of information and its role in cortical contrast processing involve the pulvinar’s ability to modulate neuronal responses. The authors proposed a model that explains the observed changes in response gain in hierarchically distinct cortical areas based on the interaction between the pulvinar and feedforward visual cortex signals.

These findings underscore the significance of computational models in advancing our understanding of pulvinar functions. Over the years, various cognitive and computational models of visual perception have put forth roles for the pulvinar. Three decades ago, Mumford (1992) proposed that the thalamus, including nuclei like the pulvinar, functions as an active blackboard that maintains an updated representation of the visual world. This enables cortical areas to be informed about relevant changes in the visual scene, requiring new neuronal computations for planning action strategies. A more recent theoretical framework, known as predictive coding, also recognizes the unique role of the pulvinar in influencing cortical processing within and between cortical areas (Kanai et al., 2015; Haarsma et al., 2021). In essence, predictive coding is a model of neuronal organization that suggests the brain constantly generates and updates an internal representation of its environment. The brain generates predictions about the state of the world based on stored sensory inputs and compares them to incoming sensory information, resulting in prediction errors. To contextualize and coordinate these predictions and prediction errors, the brain requires a regulatory mechanism that assigns “precision” to the message. Within this framework, the pulvinar could play a crucial role in processing the variability of cortical signals to modulate the transfer of feedforward and feedback information. Disruptions in this “precision-weighting” of neuronal activity have been proposed as a key mechanism underlying the pathogenesis of psychosis, including schizophrenia, which is characterized by dysfunctions in thalamocortical communication involving the pulvinar (Byne et al., 2009; Adams et al., 2013; Benarroch, 2015; Friston, 2022).

## 4. Future avenues of study and challenges

Our current understanding of the role of thalamic nuclei in vision, particularly the pulvinar and its associated pathways, still has significant gaps. It is crucial to acknowledge that a solid understanding of the anatomy is a prerequisite to comprehending the physiology. Therefore, we must continue to characterize the anatomical organization of pathways involving the lateral geniculate nucleus and the pulvinar, and explore the effects of activating or silencing their specific components. The remarkable progress in technical approaches, such as optogenetics, may prove to be valuable in this pursuit. The ability to manipulate single cell types within an intact cortico-thalamic network can provide more precise information compared to conventional silencing techniques that affect all neuron classes. This technique, combined with connectomics approaches like three-dimensional visualization of neural networks involving thalamic nuclei in transparent tissue and resting-state functional imaging, can unveil cortico-thalamic connectivity in both animal models and humans. With the knowledge we have acquired in recent years, it is crucial to refine our protocols when studying the impact of thalamic lesions on visual cognition and take advantage of technical advancements (e.g., the visualization of dLGN layers in fMRI studies). However, there are still some challenges to address. Research on the lateral geniculate nucleus may not be as glamorous as it once was, making it difficult to secure funding from granting agencies. Recording from pulvinar neurons is challenging, and only a limited number of laboratories have the expertise to do so. Furthermore, animal models are currently limited, with mice being the preferred model. However, mounting evidence suggests that the organization of transthalamic and cortico-cortical pathways in mice differs significantly from that of higher mammals.

Nevertheless, the visual thalamus remains a fascinating area of study for the next generation of neuroscientists. They now have access to powerful tools that allow for investigations into the constituent nuclei and associated pathways, enabling a better understanding of the exact role of thalamic nuclei in sensory processing and the consequences of their dysfunction. This knowledge is essential for developing tools to restore vision and holds great promise for future research.

## Author contributions

CC: Writing – original draft, Writing – review & editing. LC: Writing – original draft, Writing – review & editing.

## Funding

The author(s) declare financial support was received for the research, authorship, and/or publication of this article.

This review received financial support from CIHR (PJT-148959 & 202209PJT) and NSERC (RGPIN-2019-04982).

## Conflict of interest

The authors declare that the research was conducted in the absence of any commercial or financial relationships that could be construed as a potential conflict of interest. The author(s) declared that they were an editorial board member of Frontiers, at the time of submission. This had no impact on the peer review process and the final decision.

The author(s) declared that they were an editorial board member of Frontiers, at the time of submission. This had no impact on the peer review process and the final decision.

## Publisher’s note

All claims expressed in this article are solely those of the authors and do not necessarily represent those of their affiliated organizations, or those of the publisher, the editors and the reviewers. Any product that may be evaluated in this article, or claim that may be made by its manufacturer, is not guaranteed or endorsed by the publisher.
